# Hydrozone and Glycozone in Gastric and Intestinal Disturbances

**Published:** 1900-11

**Authors:** 


### HYDROZONE AND GLYCOZONE IN GASTRIC AND INTESTINAL DISTURBANCES.

W. H. VAIL, (*Medical Mirror*, December, 1899,) says: I have for a long time been rather enthusiastic over the value of hydrozone and glycozone in treating diseases, and can attribute much valuable assistance and extraordinary results from their use in the last few years.

I was called to treat a young man, suffering from a severe gastro-enteritis. I found him in a most serious condition, having been delirious for three days. His temperature was sub-normal, 97.6, pulse 60, respiration 16. He was greatly emaciated, atonic, had inappetence, a severe agonising pain in the stomach and intestines, at times so severe that he would sit on the edge of the bed and groan, oftentimes, yell. These attacks were always of a similar nature and occurred regularly. He was unable to take either solid or liquid food, even in small quantities without causing a return of the pain, a teaspoonful of milk being sufficient to produce it. His condition was pitiable. His cheeks were hollow, eyes congested, skin pale and sallow and his whole appearance showed the presence of intense pain.

I saw him at the end of the third week of his illness and determined that hydrozone and glycozone were the remedies indicated, therefore, I gave him, at once one-half glass of a mixture of one-half ounce of hydrozone with a little honey to one quart of water. He was somewhat disturbed for a while after the potion, but was soon relieved. I continued to administer this for some time, with only a slight improvement, but after several doses had been taken, the relief was very decided. After his nourishment, I gave one teaspoonful of glycozone in a wine glass of water. After a few doses of this, he was very much easier and, at midnight, fell asleep and slept all night not awakening until morning, the first sleep that he had had in five days. I had previously discarded all other remedies, of which there was a large number, as one after another was given with no benefit. All of the acute symptoms disappeared in a few days, at which time, he felt very much better, and he continued to improve without having a recurrence of any of his old severe symptoms. Before this, I had increased both the nature and the quantity of his food which he relished greatly. I continued the hydrozone and glycozone for a month after, to entirely reduce the inflamed condition of the mucous membrane of the gastro-intestinal tract. These two remedies have afforded me the most excellent issues many times in the treatment of gastric and intestinal disorders.

Hydrozone causes destruction to microbes, has no deleterious action upon animal cells, possesses no toxic qualities, exerts no corrosive effect upon healthy mucous membranes when used in diseases caused by germs, is a pus destroyer and a stimulant to granulating tissues.

Glycozone while not so rapid in its action as hydrozone is, nevertheless, just as sure a stimulant, and in all gastric and intestinal disorders, exerts a potent and uninjurious effect upon the diseased mucous membrane of the stomach, healing to a nicety. It is an effective oxidizing agent, has an agreeable, sweet, and at the same time, slightly acid taste, resembling lemonade. Its use produces no deleterious action on the heart, liver or kidneys.

